# Editorial

**Published:** 1865-01

**Authors:** 


### Editorial.

### DEATH FROM CHLOROFORM.

The following we copy from the *Pittsburgh Saturday Commercial*, of November 18th, 1864.

It is another of those sad warnings that have been so frequently repeated, and that Dentists seem determined to disregard.

This is the second accident of the same kind that has occured in that city within a few months. Death from the inhalation of chloroform is of very frequent occurrence, one every few weeks and, perhaps, much oftener. There are a great many fatal cases that are never reported, but enough of them are published to impress us with the fact that great care should be exercised in the administration, and that he who would attempt it should possess a very thorough knowledge of the human system, not only of its anatomical structure, but of temperament, and of physiological and pathological conditions.

Nineteen-twentieths of the dentists are totally unfit to administer chloroform; many are so from a want of attainments, others from a want of self-control in a time of great emergency. This is a very important matter; there are very few indeed, who at the time of such an emergency can retain perfect self-possession and answer the demands of the occasion. In the hands of surgeons death oftentimes occurs from the administration of chloroform.

For many operations we regard chloroform as an inestimable blessing; but for so simple an operation as the ordinary extraction of a tooth we regard its use as hardly warrantable.

We never administer chloroform without unpleasant feelings amounting often to a fear of ill results. We hope that this case together with others may serve to increase caution and care, if not the entire abandonment of chloroform in the extraction of teeth.

ANOTHER VICTIM TO THE USE OF CHLOROFORM—A CORONER'S JURY RECOMMEND THE PASSAGE OF A LAW AGAINST ITS USE—THE DENTIST ACQUITTED OF BLAME.—We are called upon to record another case of death resulting from the use of chloroform in extracting teeth. But a few brief months have passed since a lady died in the operating chair of one of our most popular dentists after the administration of an opiate intended to prevent pain whilst her teeth were being extracted. A Coroner's jury acquitted the dentist of all blame, but, if we remember aright, condemned the practice of using chloroform in such cases, as being attended with danger to the patient.

Another fatal case occured yesterday in the dental establishment of Dr. Spencer, No. 259, Penn Street. The facts as related to us are as follows: Miss Malinda Caryt, a resident of Kittanning, aged about seventeen years, had been on a visit to some relatives in this city. Yesterday, in company with her aunt, Mrs. Collins, who resides in Alleghany, she visited the office of Dr. Spencer for the purpose of having a number of teeth extracted. The ladies were ushered into the operating room by Dr. Horner, who was officiating fur Dr. Spencer, who is at present too unwell to attend personally to his professional duties. Miss Caryt stated the nature of her call and desired Dr. Horner to administer chloroform before he undertook to extract her teeth. After the usual questions as to her physical condition, the Doctor, believing her to be a fit subject, complied with her request, and administered chloric ether, which is a compound of ether and chloroform and proceeded to extract her teeth.

After he had succeeded in extracting some four or five teeth, Miss Caryt suddenly raised up in her seat and commenced to struggle in a violent manner. Discovering that something was wrong, Dr. Horner at once sent for Dr. Duncan, but before his arrival the young lady expired. Dr. Horner, immediately after proper attention had been paid to the remains of the deceased, repaired to the Mayor's office and gave himself into cnstody to await the result of the Coroner's jury.

As soon as Coroner Donaldson was apprised of the melancholy occurrence he summoned a jury, and after hearing the evidence of Dr. Duncan, Mrs. Campbell, aunt of deceased, and a young lady residing at Dr. Spencer's, the jury returned a verdict that the deceased came to her death from the use of chloroform administered at her own request, in the usual manner, by Dr. Horner, and fully exonerating him from all culpability in the matter.

The jury, however, outside of their official verdict strongly condemn the use of chloroform by the dental profession, and call upon the Legislature to pass a law inflicting heavy penalties upon all who persist in the practice. The body of the young lady was conveyed to the residence of Mr. Isaac Collins, No. 20, Main street, Allegheny.

### NERVE INSTRUMENT.

We received a short time ago from Mr. R. B. Sutton, of Guilford, Conn., some very fine nerve instruments. They are for the removal of pulps of teeth, and consist of very finely finished five sided broaches, barbed very perfectly and uniformly on their corners. They are the best finished instruments of the kind we have seen. No dentist should be without them. In the few experiments we have made with them, we find they *bring away* anything with which they come in contact. We understand they will be for sale at the dental depots.

### RE-ORGANIZATION.

We are very much pleased to learn that an effort is being made to re-organize the "Mad River Valley Dental Society." This is a move in the right direction and one that should have been made long ere this. There are good men enough in that section to sustain a good society. When the appointed time comes let every man be at his post, and put forth his influence by his personal presence, and all will be benefited by it. Our profession has been brought to its present status by associated effort, and if it is to continue its progress and development, associated effort must not be abandoned.

### ACKNOWLEDGMENT.

Within the last few months the Ohio College of Dental Surgeons has received quite a number of valuable contributions, for the Museum, Infirmary and Laboratory, so that these departments are well supplied with all needed apparatus and appliances.

Among the contributions we may notice the following, viz: From Dr. H. J. McKellops, formerly of St. Louis, now Paris, France, a large and fine collection of Anatomical preparations for illustrating the development and formation of the teeth, valued at $500.

One Dental Chair, Footstool and Spittoon, from R. W. Archer of Rochester, N. Y. Value $100.

One Dental Chair from J. S. Walter & Co. of this city. Price $50.

One two piece vulcanizer, worth $15 50, from B. T. Whitney, of Buffalo, N. Y.

Also, one three piece Vulcanizer, worth $16, from Geo. E, Hayes, of Buffalo, N. Y.

Various sums of money have been contributed for the same. purpose, by the following persons, viz: Drs. McKellops, Cameron, Keely, Taylor and others.

The encouragement thus manifested by the profession towards our Colleges, is very gratifying indeed, and we doubt not that ere long the profession will become so thoroughly impress-with the importance and advantages of College instruction for the dentist, that a general movement and effort in that direction will be made T.

### THE DENTAL CIRCULAR AND EXAMINER.

The first number of this work is upon our table, and it presents a very good appearance. It is neatly gotten up throughout. It is edited and published by Dr. B. Wood, of Albany.

Dr. W. is one of our vigorous and practical writers, and will doubtless make this a good journal, one that will result in great good to the profession. It contains thirty-two pages, and is published at $1 per year.

We hail with pleasure anything of this kind that promises usefulness and stability. T.

### THE MEETINGS.

We would direct especial attention to the notices on another page of the meetings of the Ohio Dental College Association to take place on the 21st inst., and the Mississippi Valley Dental Association to meet on the 22nd inst., both to be held at the Dental College in this city. We hope and expect a large gathering of the profession here at that time. We have assurances from a large number that they will be present.

